# Exploring the Key Influencing Factors on Teachers’ Reflective Practice Skill for Sustainable Learning: A Mixed Methods Study

**DOI:** 10.3390/ijerph191811630

**Published:** 2022-09-15

**Authors:** Zengzhao Chen, Rong Chen

**Affiliations:** Faculty of Artificial Intelligence in Education, Central China Normal University, Wuhan 430079, China

**Keywords:** sustainable learning, reflective practice, teacher education, pedagogical self-efficacy, teaching support service, personal goal-oriented

## Abstract

In 2019, the United Nations released “Education for Sustainable Development for 2030”, emphasizing that sustainable learning is an important component of education for sustainable development, as it can enable learners to master the knowledge and skills required to keep learning in a variety of circumstances. To better understand teachers’ sustainable learning within the context of education, this study used a comprehensive method combining quantitative analysis and qualitative analysis to examine the key factors that influence teachers’ reflective practice skill through educational practice for sustainable learning. A total of 349 teachers responded to the survey. Based on the quantitative results, 10 teachers were chosen for qualitative analysis. Results showed that teaching support service, peer feedback, teacher–student interaction, and personal goal orientation were found to have a significant impact on teachers’ reflective practice skill, which is beneficial for promoting sustainable learning. Interestingly, the direct impact of pedagogical self-efficacy on reflective practice skill was not observed. The following qualitative research yielded five topics on teaching support service, peer feedback, teacher–student interaction, pedagogical self-efficacy, and personal goal orientation. These topics helped to explain the results of the quantitative analysis. The findings of the proposed model were conducive to understanding the mechanism that affects teachers’ reflective practice skill as well as providing practical implications for teachers’ sustainable learning in educational practice.

## 1. Introduction

Since it was proposed in 1992, Education for Sustainable Development (ESD) has been committed to cultivating students’ concepts and abilities to deal with the future life and environment through interdisciplinary learning and cooperation. In recent years, the United Nations not only distributed the ten-year policy of “ESD for 2030” but further pointed out in SDG4 to establish inclusive and fair high-quality education to ensure that all those who are involved in learning can master the knowledge and skills needed for sustainable development through high-quality education. As a crucial component in the education for sustainable development, sustainable learning is critical in promoting the sustainable development of human society [1]. Sustainable learning not only refers to learners’ lasting retention of knowledge but also active learning, which can continuously rebuild their skills as the external environment changes [2]. Many studies have discussed sustainable learning and other related educational topics [3,4]. Some scholars have reinvented teacher education in the twenty-first century by utilizing reflective practice for sustainable learning, motivated by the notion of teachers as learners [5,6]. For instance, Bryk et al. (1999) used systematic theory to link the structural, cultural, and political dimensions of the school work environment with teacher reflection and professional learning, and they discovered that school organizational conditions such as involvement in decision making, teamwork, teacher collaboration, an environment of open trust, and transformational leadership can promote the sustainable development of teachers’ reflective practices and professional skills [7]. By using adult learning theory and teaching as a concept of reflective practice, Smylie et al. (1996) argued that reflective thinking is essential for the teachers’ sustainable learning [8]. According to a research conducted by Kwakman (2003), teaching reflection was a crucial activity for enhancing instruction and fostering student learning [9]. Teachers can obtain fresh knowledge, insights, and ideas from a variety of sources based on their teaching reflection.

Individual variables also have a significant impact on teachers’ reflection and professional development in addition to the organizational support that the external environment provides. Although it is possible to view the teaching of environmental support reflection as a crucial learning activity, not all reflection will result in a successful educational outcome since teachers may find it extremely challenging to reflect on their presumptions. Reflecting on one’s teaching can make teachers feel as though they have failed in the past, as Coburn (2004) noted, which could cause them to be hesitant when reflecting [10]. In this situation, we need to take into account the teacher reflection from the perspective of individual psychological states in order to further improve the professional learning effect of instructors. Research on the effects of psychological states on teachers’ reflection and professional development has revealed that individual elements, such as perceived control, autonomy of the teacher, and personal teaching efficacy, have an impact on teachers’ professional development and reflective practice [11]. According to the AMO theory, which holds that teachers’ professional development and teaching reflection are the joint functions of personal ability, personal motivation, and personal perceived opportunity, Runhaar et al. (2010) explored the mechanism and intervention path of teacher reflection on a personal level. They also conducted a survey of 456 teachers at the Dutch Institute of Vocational Education using this theoretical framework to look at the direct and indirect effects of motivation, aptitude, and opportunity perception on teachers’ reflection. The findings revealed a positive correlation between teaching reflection and professional self-efficacy and learning goal orientation [12].

Although there have been some studies on teachers’ reflective practice, most of them are qualitative studies that focus on the single dimension of the environmental level or individual level [13]. Few studies have systematically examined the variables and their mechanisms that influence teachers’ ability to engage in reflective practice from the standpoint of the interaction between environment and individual. Without fully understanding the key factors that affect teachers’ reflective practice skill for sustainable learning, the optimal instruction model cannot be designed, developed, implemented, and evaluated to promote the sustainable learning. Given the promise value of mixed research that combines quantitative evaluation and qualitative analysis, which may examine and explain the process of evaluation results from many angles, making the exposition of research conclusions more thorough, there is a critical need to use mixed-method approach to further our understanding of the role that reflective practice skill plays in teachers’ professional development given its great potential for developing teachers’ capacity for reflection, evaluation, and summarization.

Based on this critical issue, our research studies the relationships among the key factors influencing teachers’ reflective practice skill in the process of education.

In this article, Section 2 discusses the background, the conceptual model, and the study’s hypotheses; Section 3 describes the research methods; and Section 4 reports the research results. Then, the results are presented and discussed in Section 5, and the conclusions and the enlightenment for further research and application are proposed in Section 6 and Section 7.

## 2. Background and Hypotheses Development

### 2.1. Sustainable Learning

Sustainable learning is an educational concept based on the principles of sustainable development, which reflects people’s understanding of how to continuously learn different skills to create a more equitable and sustainable living conditions [14]. Sustainable learning is of great significance for promoting the reform of traditional education as well as realizing the education for sustainable development and building a learning society. Specifically, sustainable learning has promoted personalized learning reform and innovation; additionally, its application of “learning to learn” has promoted the popularization of “sustainable development” and “lifelong learning” in the education field, which has a significant impact on passive education and knowledge instillation in traditional education [15].

### 2.2. Reflective Practice

Reflective practice typically refers to that teachers reflect on the curriculum activities in the teaching process [16]. The effects of reflective practice on the sustainable learning of teachers’ professional skills have been covered in pertinent studies. For instance, Roth (1989) thought that, under the guidance of “reflection in action”, reflective practice is of great significance to teacher training. Teachers who can actively apply the reflective practice concept would cross the boundary between research and practice and eventually become expert teachers [17]. Teachers frequently begin a continual process of self-reflection, self-education, and self-development when they have mastered the skill of reflective practice. As a result, they frequently approach challenges from multiple points of view and actively foster their professional development by reflecting on the practice of teaching [18]. Adopting the principle of educational practice, teachers who need to identify educational difficulties and construct educational theories are lifelong learners who are capable of continuing their own education and development [19].

### 2.3. Social Cognitive Theory

As a paradigm of education that combines social context with individual cognition, the theory of social cognition has been extensively utilized in the study and practice of educational psychology [20]. According to this idea, individual behavior, psychological cognition, and environmental factors all play a role in how people interact with one another when learning new things [21]. From the perspective of social cognitive theory, both individuals and society have an impact on teachers’ ability to reflect on their practice. Reflective practice, as Watt and Lawson (2009) pointed out, takes the form of a critical reflection on one’s practice and is influenced by factors such as personal motivation, ability level, knowledge reserve, and self-efficacy. [22]. Parsons and Stephenson (2005), on the other hand, believed that supportive conditions in school organizations and social interaction with colleagues or students could stimulate teachers’ reflection [23]. Based on this, this study used social cognitive theory as the analytical framework to distinguish between the types of individual and organizational factors and to investigate the interaction between the two factors on the development of teachers’ reflective practice skill. This paper investigated the influencing factors of teachers’ reflective practice skill in conjunction with school support, peer feedback, teacher–student interaction, pedagogical self-efficacy, and personal goal orientation.

### 2.4. Teaching Support Service 

Teaching support service has been emphasized by education administrators, teachers, researchers, and the general public as an important component of cultivating teachers’ sustainable learning ability [24]. Meng (2021) investigated the factors that influence teachers’ reflection on integrated technology teaching using grounded theory and a structural equation model. The findings revealed that the factors of performance expectation, convenience, group influence, and students’ group characteristics have a direct impact on the intentional factors of Chinese teachers’ integrated technical pedagogical reflection [25]. From the perspective of school management, Steinert et al. (2019) indicated that good school leaders can not only provide a clear vision for teachers’ future career development and encourage teachers to implement curriculum innovation in teaching practice but also stimulate teachers to reflect on their practice and encourage them to challenge the old theoretical assumptions in teaching practice [26]. The impact of the principal’s leadership on teacher reflection and professional learning was investigated by Liu and Hallinger (2021). In that study, structural equation modeling (SEM) and bootstrapping tests were used to analyze the data of 1194 teachers in 64 schools. The results showed that principals had significant direct and indirect effects on teachers’ reflective practice and professional learning [27].

### 2.5. Peer Feedback

Peer feedback refers to the active interaction between teachers and teachers in educational practice [28]. Peer feedback occurs in the educational process when two or more teachers communicate and share their perspectives in order to achieve a teaching goal. In terms of the impact of peer feedback on teachers’ ability to engage in reflective practice, Thompson et al. (2019) discovered that peer feedback can lead teachers to seek guidance from more experienced colleagues, allowing them to think critically about classroom work at a deeper level [29]. Weber et al. (2018) argued that peer communication and feedback can have a positive impact on teachers’ reflective practice and professional learning because peer feedback can help teachers focus on key teaching events, which may lead them to better observe and explain their classroom management [16]. Sumantri et al. (2019) suggested that working with other colleagues can help teachers think more deeply because the reflection in a purely individual sense tends to remain at the level of reporting rather than at the level of analysis, while peer feedback can support teachers to think about their teaching productively, which can promote critical reflection, experimentation, and other types of professional development [19].

### 2.6. Teacher–Student Interaction

Teacher–student interaction is a kind of teaching method commonly used by teachers to increase the classroom atmosphere, which can build a teaching community in the teaching process and promote collaboration between teachers and students in solving educational problems [30]. In terms of the influence of teacher–student interaction on teacher reflection, dialogue interaction is the main way to promote teacher’s reflective practice skill and professional growth. By combing through the literature on teacher professional learning over the past decade, Kiemer et al. (2015) found that curriculum reform projects on teacher professional development are largely designed to move classroom practice toward a more conversational style; the aim is to provide innovative teaching strategies for teacher reflection [31]. Kazhikenova et al. (2021) studied 256 fourth- and fifth-year education students at the University of Kazakhstan and found that the dialogue and interaction between teachers and students can effectively promote the development of teachers’ reflective practice skill [32]. Through observation, investigation, interview and self-report, Clarke (2006) explored the functioning mechanisms of the teachers’ reflective practice skill. The results showed that the interaction between teachers and students and teachers promoted the reflection of teachers [33].

### 2.7. Pedagogical Self-Efficacy

Pedagogical self-efficacy usually refers to the ability to judge whether a teacher can complete a certain achievement behavior when faced with difficult tasks [34]. It is the main element of personal coping with environmental obstacles and the personal pursuit of goals in the process of sustainable learning. Since Bandura put forward the concept of self-efficacy, a series of professional self-efficacy concepts, such as scientific self-efficacy, computer self-efficacy, and creative self-efficacy, have been produced [35,36]. In the research on teachers’ career development, positive self-efficacy is believed to promote teachers’ reflection and professional learning. Schyns and Von Collani (2002) believed that self-efficacy is generally related to work conditions and positively related to different professional development activities, which has a positive effect on teachers’ reflective practice teaching [37]. Geijsel et al. (2009) pointed out that the higher a teacher’s sense of self-efficacy, the more willing the teacher to engage in teaching reflection and professional learning activities [38]. Runhaar et al. (2010) explored the relationship between teachers’ professional self-efficacy and teaching reflection and found that teachers with a strong sense of professional self-efficacy had higher expectations [12]. In addition, besides having a direct effect on teacher’s reflection, self-efficacy often plays a mediating role in the process of teacher’s reflection. For example, Loughlan and Ryan (2022) found that teacher self-efficacy mediates between the organizational support and teacher reflection [39]. Battersby and Verdi (2015) argued that professional learning communities built by peer collaboration and feedback are positively correlated with teacher self-efficacy [40].

### 2.8. Personal Goal Orientation

Personal goal orientation refers to teachers participating in teaching activities due to their enjoyment, which drives teachers’ confidence and satisfaction and constitutes the power of sustainable learning [41]. From the perspective of professional development, teacher reflection is often closely related to personal motivation [12]. As an internal psychological variable, motivation-based personal goal orientation not only has a direct impact on teachers’ reflective practice skill but acts as a mediator between the external environment and teachers’ reflection. For example, in Woerkom’s (2004) view, reflection is generally regarded as “risky”, and peer feedback often provides external support for solving this, which can indirectly promote teachers to engage in such dangerous behavior; that is, teachers with strong learning goal orientation tend to have more motivation to reflect; therefore, the perception of peer feedback may significantly increase teachers’ motivation to engage in “risky” behaviors [42]. Leithwood (2013) believed that teachers’ internalization of school goals and values is an important aspect of teacher motivation and that when teachers internalize school goals and values into goals at the level of personal development, they are more motivated to engage in teaching reflection. When the teacher’s assessment of the current environment is the same as the desired state, personal goal orientation can indirectly stimulate teachers’ reflective actions, which may affect the development of reflective practice skill [43]. Kazhikenova et al. (2021) combined quantitative and qualitative research methods and found that good personal goal orientation acts as a mediator between teacher–student interaction and teaching reflection [32].

### 2.9. Hypotheses

Based on the above arguments and evidence, this paper proposes these hypotheses:

**Hypothesis** **1** **(H1).** *Teacher–student interaction can promote teachers’ reflective practice skill.*

**Hypothesis** **2** **(H2).** *Peer feedback can promote teachers’ reflective practice skill.*

**Hypothesis** **3** **(H3).** *Teaching support service can promote teachers’ reflective practice skill.*

**Hypothesis** **4** **(H4).** *Pedagogical self-efficacy can promote teachers’ reflective practice skill.*

**Hypothesis** **5** **(H5).** *Personal goal orientation can promote teachers’ reflective practice skill.*

**Hypothesis** **6** **(H6).** *Teacher–student interaction can promote pedagogical self-efficacy.*

**Hypothesis** **7** **(H7).** *Peer feedback can promote pedagogical self-efficacy.*

**Hypothesis** **8** **(H8).** *Teaching support service can promote pedagogical self-efficacy.*

**Hypothesis** **9** **(H9).** *Teacher–student interaction can promote personal goal orientation.*

**Hypothesis** **10** **(H10).** *Peer feedback can promote personal goal orientation.*

**Hypothesis** **11** **(H11).** *Teaching support service can promote personal goal orientation.*

## 3. Methodology

### 3.1. Quantitative Phase

#### 3.1.1. Participants

In the quantitative research stage, this research took the city as the unit, and teachers from Hunan, Guangdong, Beijing, and Hubei Provinces were selected for the study. A total of 349 valid questionnaires were collected. Among these effective questionnaires, 121 (34.7%) were male teachers, and 228 (65.3%) were female teachers. After obtaining permission from school administrators and instructors, this research conducted an assessment by distributing questionnaires on-site towards the end of the semester. Before the evaluation, the researcher told all teachers the purpose of the evaluation, that participation in the evaluation was completely voluntary and anonymous, and that the evaluation would not be linked to the year-end assessment and performance appraisal.

#### 3.1.2. Instruments

This study used a self-reported questionnaire survey method. The latent variables of the research model include teaching support service (TSS), peer feedback (PF), teacher–student interaction (TSI), pedagogical self-efficacy (PSE), personal goal orientation (PGO), and reflective practical skills (RPS). The items of the observed variables were all grounded on the existing mature research scales. Among them, TSS was adapted from the theory of adult learning in the school organization support scale [44]. PF was adapted from the reflective capacity scale [45]. TSI was adapted from the Reflective Practice Questionnaire [38]. PSE was adapted from the theory of career self-management in the career self-efficacy scale [46]. PGO was adapted from the goal orientation subscale [47]. RPS was adapted from the reflective practice subscale [48]. The six scales cited in the study were originally in English. Considering that not all of the test participants are English teachers, and there are certain language barriers in reading the English questionnaire, the study translated the original English questionnaire into a Chinese questionnaire. Two doctoral students in the field of educational technology and a post-doctoral student in the field of educational psychology parallel translated the survey items with committee reconciliation as a means for pre-assessing the translated draft. Afterward, to increase the expert validity of the scale, the questionnaire was handed over to another pedagogical scholar with a bilingual teaching background. This study used Likert’s five-point scale. Among them, “1” means completely disagree, “2” means disagree, and so on until “5” means completely agree (Table 1). In this study, structural equation modeling (SEM) was used to estimate and verify the influencing factors of the relevant aspects [49], and spss27.0 and AMOS23.0 tools were used for analysis.

#### 3.1.3. Data Collection and Analysis

In the quantitative research phase, structural equation modeling (SEM) was used to estimate and validate the influencing factors and pathways, and SPSS27.0 (SPSS Inc., Chicago, IL, USA) and AMOS23.0 (SPSS Inc., Chicago, IL, USA) were used to analyze. SPSS 27.0 was used to perform a descriptive statistical analysis of the collected receipts and to estimate the reliability of the scale by calculating Cronbach’s alpha. In addition, using AMOS23.0 to carry out confirmatory factor analysis, construct stable factor structure, further evaluate the reliability and validity of the research model, and analyze the reliability of the structural equation model by calculating fitting index coefficients, we finally determined the environmental level of factor variables and personal level of factor variables on teachers’ reflective practice skill path. The maximum likelihood estimation (ML) method was used to verify the proposed research model.

### 3.2. Qualitative Phase

#### 3.2.1. Participants

In order to further explore and interpret the results of the quantitative phase, the research also used the form of in-depth interviews to conduct qualitative investigations [50]. This research invited 10 teachers to participate in the qualitative interview research. In the end, a total of 49 valid answers were obtained, and almost every teacher expressed their feelings after participating in educational practice. 

#### 3.2.2. Instruments

The qualitative interview protocol was grounded on the initial results of quantitative research. The qualitative analysis mainly includes five dimensions: teaching support service, peer feedback, teacher–student interaction, pedagogical self-efficacy, and personal goal orientation. In order to increase the content validity of the questionnaire, a pilot test was conducted among two teachers and two experts with 5 years of teaching and research experience, and the questions included in the questionnaire were finally determined. One question investigated teachers’ perceptions of teaching support service (i.e., “In the course of your teaching practice, do you think the organization support service provided by the school has any effect on your teaching reflection? Please combine your own actual teaching experience to talk about specifics”). A question surveyed teachers’ perceptions of peer feedback (i.e., “In the course of your teaching practice, do you think the evaluation feedback among colleagues would have an impact on your teaching reflection? Please combine your own actual teaching experience to talk about specifics”). One question explored teachers’ views on teacher-student interaction (i.e., “In the process of your teaching practice, do you think the interaction with your students would have an impact on your teaching reflection? Please combine your own actual teaching experience to talk about specifics”). One question explored teachers’ views on pedagogical self-efficacy (i.e., “In the process of your teaching practice, do you think that having good teaching ability would have any influence on teaching reflection? Please combine your own actual teaching experience to talk about specifics”). One question explored teachers’ views on personal goal orientation (i.e., “In the process of your teaching practice, do you think the challenging teaching tasks would have any impact on your teaching reflection? Please combine your own actual teaching experience to talk about specifics”). 

#### 3.2.3. Data Collection and Analysis

Data were collected in June 2022. All responses that teachers participate in were voluntary and followed strict ethical requirements. The research conducted interviews with teachers in different schools, recorded the interview data with a voice recorder, and then transcribed the voice data. In addition, the research adopted the coding method of grounded theory and NVIVO11.0 software (QSR International, Burlington, MA, USA) to analyze the data. In the open coding stage, different research topics were divided according to semi-structured interview scenes, and the dialogue was cut into situational dialogue segments according to the dialogue themes, and the segments of the conversation are refined and structured to form a small number of topics; in the selective coding stage, based on the transcribed corpus combined with the types of interview topics, the results of different groups of interview data were extracted to determine the logical relationship between the themes and the topics Finally, conclusions were written based on the category relationship, which can help further explain the results produced by the current study.

### 3.3. Research Model

**Figure 1:**
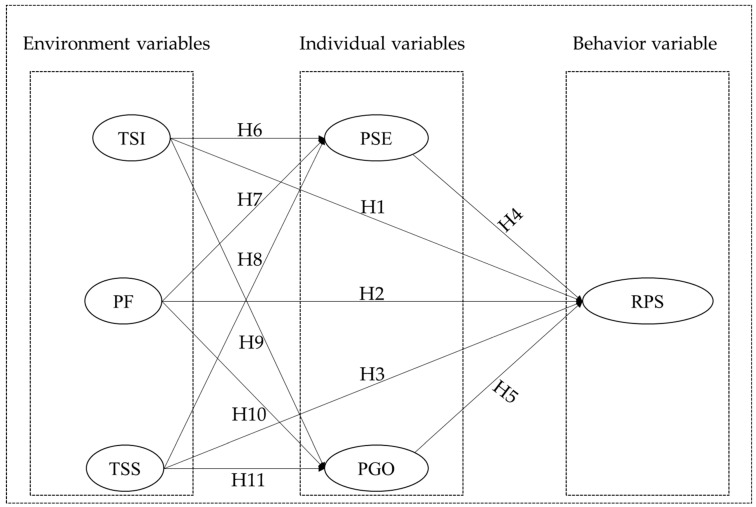
Basic research models and assumptions.

According to the above analysis, the development of teachers’ reflective practice skill is not only influenced by the organizational support at the environmental level and the inner psychology at the individual level but also by the common influence of both. Specifically, in addition to teaching support service at the environmental level, peer feedback, and teacher–student interactions, besides the direct influence of individual pedagogical self-efficacy and personal goal orientation on the development of teachers’ reflective practice skill, the environmental teaching support service, peer feedback, and teacher–student interaction also have some indirect effects on the development of teachers’ reflective practice skill through individual pedagogical self-efficacy and personal goal orientation. Based on the analysis of the interaction mechanism among the variables in the above theoretical frameworks, the proposed model and hypothesized relationships of this study are as follows, reflected in Figure 1:

## 4. Results

### 4.1. Quantitative Phase

Based on structural equation modeling (SEM), this study analyzed the measurement and structural models by using Amos and SPSS. Generally speaking, the SEM includes the measurement model and the structural model. Reliability, convergent validity, and discriminant validity were the common indicators to evaluate the measurement model, and the structural model was usually used for estimating the explanatory verification of the model by the path coefficient.

#### 4.1.1. Measurement Model Analysis

According to Chin (1998) and Hair et al.’s (2009) criterion, the construct validity was examined by three principles of convergent validity. First, the factor loading (λ) should be higher than 0.5, and their *p*-value should be significant. Second, the composite reliability (CR) should be greater than 0.6. Third, the average variance extracted (AVE) should be greater than 0.5 [51,52]. In this study, all factor loadings were greater than 0.6, and all items were between 0.62 and 0.87. The CR of all constructs showed high results: the range was between 0.781 and 0.862. Most average variance extracted (AVE) values were larger than 0.5 and between 0.428 and 0.610. The Cronbach’s α for all projects was higher than 0.7, and all items were between 0.774 and 0.859, indicating a high degree of confidence, which confirmed the good convergent validity of this measurement model (Table 2). Moreover, Table 3 shows that the square roots of the AVEs (which are given diagonally with bold letters) of constructs were mostly greater than the correlation between them, which means that the discriminant validity of the measurement model is adequate in this study.

#### 4.1.2. Structural Model Analysis

In terms of structural model analysis, this study used the chi-square value by the degrees of freedom (χ^2^/df), GFI, the comparative fit index (CFI), the Tucker–Lewis Index (TLI), the standardized root mean square residual (SRMR), and the root mean square error of approximation (RMSEA) to assess the model fit [53]. According to the suggestion by Meyer et al. (2016), when χ^2^/df was smaller than 3, GFI and AGFI were larger than 0.8, CFI and TLI were larger than 0.9, and SRMR and RMSEA were smaller than 0.08, the model demonstrated a good fit [54]. Table 4 lists the fitness of all model parameters in this study, and all the fitness indicators reached Meyer’s recommended values, indicating that they were within the acceptable range, so the results of SEM showed that the hypothesized model could fit the data well.

#### 4.1.3. Path Relationship Analysis

**Figure 2:**
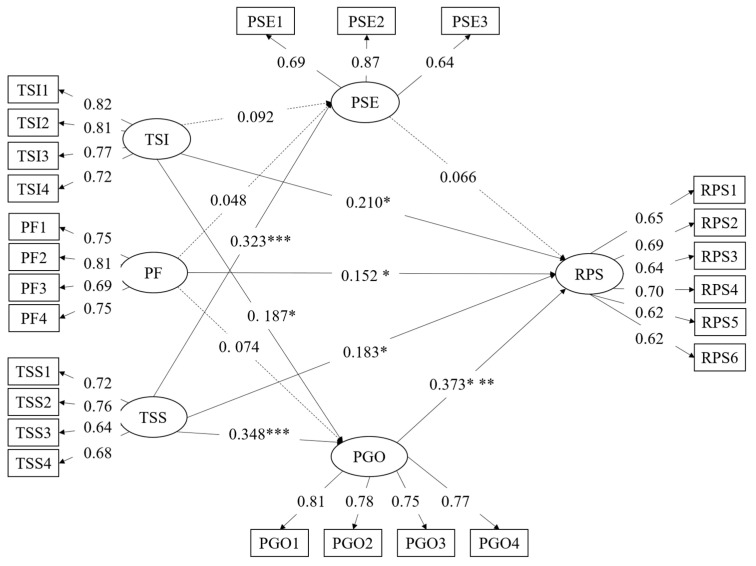
The structural model diagram of teachers’ reflective practice skill. Note: * *p* < 0.05, *** *p* < 0.001.

In order to verify the research hypotheses, a structural equation modeling analysis was conducted. Figure 2 shows the path coefficients of standardized regression weights β values and *p*-values. As shown in Table 5, seven path coefficients achieved significant results. Specifically, among them, teaching support service (β = 0.18, Z = 2.45, *p* < 0.05), peer feedback (β = 0.15, Z = 2.15, *p* < 0.05), teacher–student interaction (β = 0.21, Z = 2.78, *p* < 0.05), and personal goal orientation (β = 0.37, Z = 5.52, *p* < 0.001) had significant effects on teachers’ reflective practice skill, collectively accounting for 56% of R^2^, and the predictive effect of pedagogical self-efficacy (β = 0.07, Z = 1.17, *p* > 0.05) on teachers’ reflective practice skill was not significant. In addition, the teaching support service (β = 0.32, Z = 3.79, *p* < 0.001) had a significant positive effect on pedagogical self-efficacy. However, teacher–student interaction (β = 0.09, Z = 1.02, *p* > 0.05) and peer feedback (β = 0.05, Z = 0.55, *p* > 0.05) had no statistical effect on pedagogical self-efficacy. In addition, teaching support service (β = 0.35, Z = 4.35, *p* < 0.001) and teacher–student interaction (β = 0.19, Z = 2.22, *p* < 0.05) had a significant effect on personal goal orientation, collectively accounting for 27% of R^2^. The values of R^2^ were between 0.27 and 0.56, which shows that the model has the good explanatory ability. In other words, H1, H2, H3, H5, H8, H9, and H11 in this study were supported.

#### 4.1.4. Mediating Effect Analysis

From the above analysis, we can see that teacher–student interaction and school support services have a significant positive impact on personal goal orientation, and personal goal orientation has a significant positive impact on teachers’ reflective practice skill. Therefore, teacher–student interaction and school support services may have a potential mediating effect on teachers’ reflective practice skill. To further test for the existence of an in-effect, this study used Bootstrap 2000 time sampling and ran hypothesis tests with a confidence interval of 95% to test the mediating effect of personal goal orientation between teacher–student interaction and school support service and teachers’ reflective practice skill. The results are shown in Table 6. According to the results, there is a statistically significant mediating role of personal goal orientation in the process of school support services affecting teachers’ reflective practice skill, but in the process of teacher–student interaction affecting teachers’ reflective practice skill, there is no statistical mediation.

### 4.2. Qualitative Phase

The main emergent themes related to teachers’ reflection on teaching support service, peer feedback, teacher–student interaction, pedagogical self-efficacy, and personal goal orientation were as follows: (1) organizational incentives and feedback provided by school support; (2) key events and democratic atmosphere constructed by peer evaluation; (3) reflective dialogue and willingness to reflect driven by teacher–student interaction; (4) reflective disregard and one-sided reflection brought about by high pedagogical self-efficacy; and (5) professional learning and active perception of reflection opportunities driven by personal goal orientation, specifically as shown in Table 7.

#### 4.2.1. The Impact of the External Environment

##### Organizational Incentives and Feedback Provided by School Support

The support conditions provided by schools can stimulate teachers to reflect on their practice using organizational incentives and promote their enthusiasm for reflection to improve the quality of education. As the teacher said, “In recent years, our school attached great importance to the cultivation of teachers’ awareness of teaching reflections. School leaders tried to promote teaching reform and innovation by stimulating teachers’ reflections. For example, the school provided us with various research opportunities and employ experts through project guidance to provide personalized guidance for our teaching reflection. I thought these measures have created a positive reflection atmosphere for us.” (Teacher 2). 

Leaders’ encouragement of teachers’ courage to carry out teaching reform would promote teachers’ internal motivation in the teaching process and stimulate their teaching willingness to carry out practical innovation. This might also explain the mediating effect of personal goal orientation in the process of the influence of instructional support services on teachers’ reflective practice skill.

Feedback from schools could help teachers focus on the scope of reflection and improve the quality and level of reflection. According to the results of the interviews, “the more importance schools attached to the feedback of teachers, the more willing teachers would be to reflect and improve teaching effect. The school organized various school-based curriculum teaching and research activities to provide a broad stage for my reflective practice. In the educational practice, School leader’s feedback could not only improve my reflection process in the height and depth, but let me focus on the next work direction…” (Teacher 6).

##### Key Events and Democratic Atmosphere Constructed by Peer Evaluation

As for the influence of peer feedback on reflective practice skill, the teachers mostly reflected that peers helped them to establish the framework of classroom reflection through sorting out key events as well as a democratic atmosphere of communication to enhance the depth of reflection. For example, some teachers explained, “Every Time I received a request from the head of the teaching group to write a teaching reflection, I didn’t know how to write. Sometimes, even when I was writing alone, I would only give a brief description of what happened in the teaching. I didn’t know how to write or how to reflect. However, the situation in open classes was much better. Before taking open classes, I would find a few experienced teachers to help me. Every time I was instructed by these experienced teachers, I could have a holistic and critical grasp of the curriculum, and I could think about what I was not doing well enough and how to avoid these situations in the formal class.” (Teacher 1); “The framework of critical events provided by experienced teachers helps me to explain why I use this method of teaching and not that method of teaching at a particular time, and how I adjust the pace of teaching, teaching strategies, and teaching planning.” (Teacher 3).

The free exchange and communication of the teaching community could deepen the reflection of teachers and further promote the development of their critical thinking: “Personally, when I was in my last job, I didn’t usually like to do reflection, because reflection often meant not only finding fault with my teaching, but exposing my teaching to others, so most of the time I just wrote a little bit of my reflection. But in this new unit, I found that the atmosphere in the current teaching and research group is particularly good. My colleagues often conduct some benign competitions and evaluations among themselves. My colleagues could comment on them equally, and I had learned a lot from their comments in many cases. In addition to being able to reflect deeply on my teaching, I could also sometimes critically accept, reflect on and reprocess others’ comments.” (Teacher 4).

From this aspect, it could be seen that a good communication atmosphere among peers is a favorable condition for promoting teachers to conduct in-depth reflection, while an environment of suspicion and vicious competition could not effectively promote teachers’ teaching reflection.

##### Reflective Dialogue and Willingness to Reflect Driven by Teacher–Student Interaction

The dialogue reflection model established by teacher–student interaction was beneficial to the development of teachers’ reflective practice skill: “In addition to thinking about how the teaching process is influenced by me, at the same time, I also need to think about how teaching is influenced by students from the perspective of students, and how my interaction with students ultimately affects the direction of teaching. In the reflective dialogue established through the teacher-student interaction, I had to abandon the reflective summary that I used to carry out only from my perspective, but needed to listen to students’ feelings, perceive their positions in the teaching process, and then establish this interactive reflective process.” (Teacher 3).

Thus, teacher–student interaction, besides building a constructive and interactive audience and establishing an exchange environment of free thought and equal interaction, also helped teachers to present and solve practical problems related to students’ practical experience and to rethink and understand standardized and stereotyped summaries of teaching experiences.

A positive intention of reflection was brought about by teacher–student interaction. Good teacher–student interaction in the teaching process was conducive to the promotion of teachers’ willingness to reflect. As some teachers reported, “I reflected on the interaction that takes place in the classroom not just to fulfill the task of teaching, but to continuously improve teaching effect. Sometimes I reflected on the interaction process and found that in order to improve the quality of the course. I also needed to adjust my teaching style according to the actual situation of the students’ interaction.” (Teacher 4); “The focus on the students made me constantly think about the advantages and disadvantages of the teaching process. When students actively cooperate with my teaching work and actively interact with my teaching in class, I am more willing to think about how to attract more students to participate in the teaching interaction, of course, when I reflect on the lack of interaction between students and my feeling, I would also adjust the teaching strategy in time.” (Teacher 1). 

It could be seen that the positive teacher–student interaction promotes teachers’ awareness of reflection and stimulates their willingness to improve their teaching effect through reflection.

#### 4.2.2. The Impact of the Internal Psychology

##### Reflective Disregard and One-Sided Reflection Brought about by High Pedagogical Self-Efficacy

High pedagogical self-efficacy would lead teachers to neglect the value and importance of teaching reflection and neglect the act of teaching reflection, which would affect the development of reflective practice skill: “In my opinion, when a teacher already had good professional skills, there was no need to reflect too much on teaching. For example, I seldom saw senior teachers around me writing reflection logs. Moreover, as far as daily teaching reflection was concerned, the effect of improving teaching skills by writing teaching reflections after class was very limited. I didn’t have much self-reflection, and even if I did, I only wrote a little symbolically to hand over” (Teacher 8). 

The results of these interviews suggested that the more highly qualified the teacher thinks he or she is, the more likely he or she is to neglect to reflect on his or her teaching. Therefore, it was more difficult to promote their professional development through teaching reflection.

When teachers were too confident in their teaching ability, they tended to ignore the feedback from their peers and leaders, which leads to a situation of unilateral teaching reflection from a personal perspective. As one teacher said, “As a teacher with nearly 20 years of teaching experience, I considered myself to have a good professional ability to deal with all kinds of teaching events. In all my years of teaching practice, my class had never experienced any major teaching accidents. And when I handled these things, I didn’t need to ask for advice from others. Usually, I just needed to reflect a little bit on what happens in the classroom after class.” (Teacher 7).

These findings suggested that teachers believe that they can cope with difficult situations and meet the requirements of teaching reflection. They would probably only reflect on teaching practice from their perspective, and they often ignored feedback from their colleagues or their managers, which makes it difficult to reflect on teaching at a holistic level in the light of the advice provided by others.

##### Professional Learning and Active Perception of Reflection Opportunities Driven by Personal Goal Orientation

When teachers set personal teaching goals to improve their abilities and accomplish new, more complex tasks, they were more likely to engage in professional learning through teaching reflection: “As far as self-evaluation was concerned, I had high requirements for myself. For example, when I encountered certain complicated tasks or setbacks in teaching, I would not give up easily. These were all important steps to improve me and become a good teacher. I would think about what caused these situations and reflect on what I should do to avoid these things in the following teaching practice.” (Teacher 2); “As for me, apart from normal classes, I would often take part in various extra-curricular activities to improve my professional knowledge and teaching skills, such as taking part in various teaching competitions and research projects. By taking part in these professional learning activities, I could get a lot of guidance and feedback on my teaching, regardless of whether the comments or suggestions were pertinent or sharp. These were all essential parts of my reflection on my teaching and improving my professional skills.” (Teacher 6).

Personal goal orientation was beneficial to promote teachers to actively perceive the opportunity of reflection and reflect on various teaching elements in time. As some teachers said, “In daily teaching practice, I would seize every opportunity to improve my teaching skills, I would try my best to reflect on all kinds of issues related to teaching, whether it was guidance given to me by the school leadership or students in the classroom after-school reaction, I would seriously think about this valuable part, whether it was what I have done well or lack of place.” (Teacher 3); “I might have a high personal requirement of their own, I attached great importance to get the opportunity to improve themselves from others, whenever others evaluated my teaching, I would try to learn from their opinions, and reflected on the teaching value in this regard.” (Teacher 4). 

These results illustrated the mediating effect of personal goal-oriented teaching support service on the development of teachers’ reflective practice skill.

## 5. Discussion

Up to now, there were few literature materials to carry out empirical analysis on the key factors and action paths that affect the development of teachers’ reflective practice skill from the interaction of external environment and individual psychology. This study was an attempt to explore the core factors and mechanisms that influence the development of teachers’ reflective practice skill in the practice of Chinese education reform and to fill the gaps in the existing literature; thus, it could provide experience support for the research path and application practice of promoting teachers’ professional development with reflective practice skill. This research adopted the method of combination of quantitative and qualitative research to analyze the core factors and the mechanism of the influence on the development of teachers’ reflective practice skill. In this quantitative study, structural equation modeling (SEM) was used to explore teacher–student interaction, peer feedback, and school support services in the external environment, and the individual psychological level of pedagogical self-efficacy and personal goal orientation in the teacher’s reflective practice of the role of the path. Then, combined with the results of the quantitative research, the qualitative analysis method was used to explain the mechanism of the results obtained from the structural equation model.

It was found that the external environment level of teaching support service has a significant positive impact on teachers’ reflective practice skill. According to the results of the study, when teachers perceived the support services provided by school organizations in their teaching practice, they would show more reflective practice. On the contrary, when school organizations only provided few support services for teachers’ teaching practice, teachers were less willing to reflect on their teaching actions, and their reflective practice skill was reduced. This finding supported Steinert’s et al. (2019) research, which showed that organizational support provided at the school level can significantly affect teachers’ ability to engage in reflective practice [26]. The result also supported Meng’s (2021) finding suggesting that convenience, group influence, and students’ group characteristics have positive effects on teachers’ reflective intention and reflective behavior [25]. In addition, the study also responded to Liu and Hallinger’s (2021) findings that leadership support in school organizations has a significant direct and indirect impact on teachers’ reflective practice and professional learning [27]. Through the qualitative interviews, we could see that the support services provided by school organizations can influence teachers’ reflective practice skill development from the aspects of organizational motivation and feedback. This means that schools should focus on providing teachers with a variety of incentives and facilities for reflective teaching.

According to the research results, the external environment of peer feedback could significantly affect the development of reflective practice skill of teachers. The finding suggested that teachers who receive frequent feedback from colleagues about their teaching are more likely to reflect on their teaching. This finding was in line with previous research by Weber et al. (2018), which showed that peer communication and feedback can have a positive impact on teachers’ reflective practice and professional learning, and peer feedback could help teachers pay more attention to the key teaching events through reflection rather than the whole teaching process [16]. Through further qualitative analysis, it could be seen that equal and free communication among colleagues could provide a good guiding framework and reflection atmosphere for teachers to further enhance the depth and breadth of reflection. Combined with the above findings, this study suggested that in order to promote teachers to better reflect on teaching practice, the school group should try to establish a democratic teaching evaluation exchange situation and a constructive guiding framework for classroom reflection.

The results showed that teacher–student interaction in the external environment has a significant positive impact on teachers’ reflective practice skill. This finding was consistent with previous studies indicating that teacher–student interaction in classroom teaching has a positive predictive effect on teachers’ reflective practice teaching [55]. Mohan and Chand (2019) argued that teachers can provide rich material for reflection when they share their teaching experiences with students and engage in dialogue, which can promote teachers from the perspective of learners to rethink teaching and thus promote the professional growth of teachers [56]. In addition, this study extended Clarke’s (2006) research, demonstrating quantitatively that teacher–student interaction has a positive and significant effect on the development of teachers’ reflective practice skill in the process of teaching practice [33]. Through the qualitative interview, we could see that the reflective dialogue and reflective intention obtained from the interaction between teachers and students can promote teachers to view the teaching experience from the perspective of learners and promote intrinsic motivation in the process of reflection so that they can actively, continuously, and wholeheartedly engage in the reflective teaching practice. This also means that in the process of their teaching practice, teachers should pay attention to strengthening communication with students and try to rethink teaching from the perspective of learners.

Interestingly, the individual psychological level of pedagogical self-efficacy had no significant impact on teachers’ reflective practice skill. This finding was inconsistent with previous studies on teachers’ teaching reflection and individual perceptions of their ability to complete tasks [38]. The existing research showed that teachers’ self-efficacy has a significant correlation with their reflective practice skill [12]. The results of qualitative interviews showed that when teachers have a high personal assessment of their teaching ability, they tend to neglect to think deeply about the feedback from their peers and school leaders, and neglecting the significance and function of teaching reflection for their professional development might reduce the development of their reflective practice skill. At the same time, this study provided useful evidence for further understanding the factors that affect the development of teachers’ reflective practice skill and their mechanism of action. In the process of studying the influence of teachers’ reflective practice teaching, one surprising result was that there were almost no non-significant outcome factors, and almost all of the studies reported positive effects; although this was an obvious positive conclusion, it did not let us have an in-depth understanding of the impact on teachers to carry out in-depth teaching reflection.

The study also found that the individual psychological level of goal-oriented teachers can positively affect the development of reflective practice skill. The finding supported Barrett’s et al. (2020) research, which suggests that reflection is a goal-oriented activity aimed at improving practice, and the personal goal orientation for a teacher’s reflective practical abilities has the positive promotion function [57]. The finding also supported Daumiller’s et al. (2019) study that there is a significant positive correlation between teachers’ goals and their reflective practice teaching [58]. In addition, from the results of the study, personal goal orientation was the most important factor that affects the development of teachers’ reflective practice skill in the process of classroom teaching. Personal goal orientation not only had a direct impact on reflective practice skill, but it also played an indirect role as an intermediary variable in the process of school support service affecting teachers’ reflective practice skill. The qualitative results further explained the conclusion that teachers with a high level of personal goal orientation would actively perceive various opportunities for reflection.

## 6. Conclusions

Considering the crucial of reflective practice skill for sustainable learning, it is important to understand the relationship between teachers’ reflective practice skill and their key influencing factors in educational practice. The purpose of this study was to explore the impact of individual and organizational factors on teacher learning. We used social cognitive theory to explain teachers’ reflective practice skill through external environment dimension and individual psychological dimension to distinguish specific types of factors. Considering that people have different perceptions of reality, their reflective practices were mainly driven by perceptions, the central question of this study is, “How can the development of teachers’ reflective practice skill be explained by the interaction of environmental-level teaching support service, peer feedback, and teacher–student interaction, as well as individual-level pedagogical self-efficacy and personal goal orientation?” Overall, the final results meet most of the expectations of previous theoretical assumptions. Specifically, on the one hand, this study proved that teachers’ reflective practice skill is not only influenced by teaching support service, peer feedback, and teacher–student interaction in the context but also by the individual psychological level of the impact of personal goal orientation. On the other hand, this study proved that the development of teachers’ reflective practice skill was shaped by both individual and organizational factors. These results suggested that future research on teachers’ reflective practice skill and professional development should not only consider the influence of individual factors from external environment or individual psychology but also consider the interaction between different factors.

This study provided some enlightenment for educational managers and educators to design, develop, and implement reflective practice curriculum and teaching activities to promote the development of teachers’ reflective practice skill. For example, in the aspect of teaching support service, schools should attach importance to the training of teachers’ teaching reflection consciousness, such as the way that school leaders organize and carry out competitions to stimulate teachers’ reflection, promote their willingness to reflect, and improve their continuous reflection. In the area of peer feedback, there was a strong need to design more opportunities and activities for teachers to communicate, discuss, and collaborate so that teachers can be aware of peer feedback and teaching guidance promptly. In the aspect of teacher–student interaction, educational managers should create interactive teaching situations to promote teachers’ reflective motivation through active teacher–student interaction and then improve the ability of teachers to reflect on teaching from the perspective of learners. With regard to personal goal orientation, teachers could be provided with a variety of personal perceptions through the creation of challenging and more complex teaching tasks. In short, to better promote teachers’ reflective practice skill and further promote their professional development, school managers should not only strengthen the supporting conditions of providing teaching support service for teachers, peer feedback, and communication with students but also pay attention to stimulating teachers’ goal orientation on an individual psychological level.

## 7. Limitations and Future Directions

In addition to the above research results, this study has some limitations. In terms of research content, this research only used the structural equation model to examine several key factors that affect sustainable learning. Future research can continue to add other related factors that may affect teachers’ sustainable learning, such as student style, teaching strategy, and classroom atmosphere. In terms of research methods, the data in this study were all derived from cross-sectional questionnaires; the significant differences in teachers’ gender, years of service, study, and school location were not tested. In the future, longitudinal data can be used to explore the causal relationship between the sustainable learning of teachers and their key variables in educational practice and used to explore the differences in teacher titles, gender, years of service, and majors. In terms of research objects, this research was only a survey of Chinese school teachers. The universality of the research results needs to be improved. In the future, further cross-regional comparative research will help understand the school and cultural differences in educational practice.

## Figures and Tables

**Table 1:** Items of each dimension.

Aspect	Indicators	Items
Teaching support service	TSS1	The school assists teachers in trying out new teaching evaluation methods.
	TSS2	The school guides teachers to discuss new ideas related to professional development.
	TSS3	The school helps teachers think about the new experiences they have gained at work.
	TSS4	The school guides teachers on the summary of teaching.
Peer feedback	PF1	As I reflected on my teaching with others, I realized something I hadn’t thought about before.
	PF2	When I reflect on teaching with others, I develop a new perspective.
	PF3	I have found that reflecting on my teaching with others can help me to solve the problems I may encounter.
	PF4	When I reflect on my teaching with others, I gain new insights.
Teacher–student interaction	TSI1	In interacting with my classmates, I can realize that my pre-existing beliefs influenced the interaction.
	TSI2	In my interactions with classmates, I consider how my thoughts and feelings affect the interaction.
	TSI3	In my interactions with my classmates, I can recognize how my classmates’ pre-existing beliefs have influenced my interactions.
	TSI4	In the interaction with the students, I can realize that the students’ thoughts and feelings influenced the interaction.
Pedagogical self-efficacy	PSE1	I believe that I can effectively deal with the unexpected incidents.
	PSE2	I think I’m ready to meet most of the requirements of teaching.
	PSE3	My past teaching experience prepared me for the future of my career.
Personal goal orientation	PGO1	I like the challenging tasks in teaching where I can learn new skills.
	PGO2	I would like to choose a challenging teaching task; I can learn a lot from it.
	PGO3	I often look for opportunities to develop new skills and knowledge.
	PGO4	I often read books related to education to improve my ability.
Reflective practical skills	RPS1	I can reflect on the relationship between teaching practice and student learning.
	RPS2	I can work hard to promote the study of all the students.
	RPS3	I can look for ways to relate new concepts to students’ prior knowledge.
	RPS4	I have a real curiosity about the effectiveness of teaching practice to carry out the corresponding teaching experiments and research.
	RPS5 RPS6	I can engage in constructive criticism of my teaching. I try to find alternative ways to express ideas and concepts to students.

**Table 2:** Construct convergent validity.

Aspect		Standardized Loading	CR	AVE	Cronbach’s α
Teacher–student interaction	TSI1	0.82	0.862	0.610	0.859
	TSI2	0.81			
	TSI3	0.77			
	TSI4	0.72			
Peer feedback	PF1	0.75	0.838	0.564	0.835
	PF2	0.81			
	PF3	0.69			
	PF4	0.75			
Teaching support service	TSS1	0.72	0.794	0.492	0.794
	TSS 2	0.76			
	TSS 3	0.64			
	TSS 4	0.68			
Pedagogical self-efficacy	PSE1	0.69	0.781	0.548	0.774
	PSE2	0.87			
	PSE3	0.64			
Personal goal orientation	PGO1	0.81	0.860	0.605	0.859
	PGO2	0.78			
	PGO3	0.75			
	PGO4	0.77			
Reflective practical skills	RPS1	0.65	0.817	0.428	0.817
	RPS2	0.69			
	RPS3	0.64			
	RPS4	0.70			
	RPS5	0.62			
	RPS6	0.62			

Note: AVE, average variance extracted; CR, composite reliability.

**Table 3:** The discriminant validity of the measurement model. Bold limbs are the average variance extracted (AVE).

Variables	TSI	PF	TSS	PSE	PGO	RPS
TSI	**0.781**					
PF	0.616	**0.751**				
TSS	0.538	0.487	**0.701**			
PSE	0.296	0.262	0.396	**0.740**		
PGO	0.420	0.358	0.484	0.213	**0.778**	
RPS	0.579	0.522	0.578	0.320	0.619	**0.654**

**Table 4:** Test of hypotheses in the structural model.

Fit Index	Recommended Level of Fit	Proposed Research Model
CMIN/DF	<3	2.280
SRMR	≤0.08	0.048
RMSEA	≤0.08	0.061
GFI	≥0.8	0.873
AGFI	≥0.8	0.841
TLI	≥0.9	0.900
CFI	≥0.9	0.913

**Table 5:** Test of hypotheses in the structural model.

No.	Hypothesized Relation	Standardized Estimates	Test Results
H1	TSI→RPS	0.210 *	Supported
H2	PF→RPS	0.152 *	Supported
H3	TSS→RPS	0.183 *	Supported
H4	PSE→RPS	0.066	Unsupported
H5	PGO→RPS	0.373 ***	Supported
H6	TSI→PSE	0.092	Unsupported
H7	PF→PSE	0.048	Unsupported
H8	TSS→PSE	0.323 ***	Supported
H9	TSI→PGO	0.187 *	Supported
H10	PF→PGO	0.074	Unsupported
H11	TSS→PGO	0.348 ***	Supported

Note: * *p* < 0.05, *** *p* < 0.001, TSI, teacher–student interaction; PF, peer feedback; TSS, teaching support service; PSE, pedagogical self-efficacy; PGO, personal goal orientation; RPS, reflective practical skills.

**Table 6:** Mediating effect among variables.

Hypothesized Effects	Estimate	*p*-Value	Lower	Upper
Total indirect TSI > PGO > RPS	0.059	0.074	−0.007	0.161
Total effect TSI > RPS	0.237	*	0.055	0.454
Total indirect TSS > PGO > RPS	0.123	**	0.053	0.231
Total effect TSS > RPS	0.297	**	0.119	0.502

Note: * *p* < 0.05, ** *p* < 0.01. Outcome tests for mediation were performed in Amos 23.0. (Confidence interval = 95%, samples = 2000).

**Table 7:** Qualitative Phase Coding Analysis.

Category	Main Category	Corresponding Category	Category Connotation
External factors	Organizational incentives and feedback provided by school support	Leadership encouragement	Providing organizational support at the school level
		School management	Providing a clear vision for teachers’ future professional development
		Principal Support	Encouraging teachers to innovate courses in teaching practice
	Key events and democratic atmosphere constructed by peer evaluation	Colleague Guidance	Working with other colleagues helps teachers think further
		Learning experience	Getting guidance from more experienced colleagues
		Professional advice	Helping teachers focus on key teaching events
	Reflective dialogue and willingness to reflect driven by teacher–student interaction	Teacher–student dialogue	The dialogue and interaction between teachers and students can effectively promote teachers’ reflective practice ability
		Teaching participation	Teachers actively participate in the course teaching
		Classroom feedback	Teachers give feedback on students’ learning process
Internal factors	Reflective disregard and one-sided reflection brought about by high pedagogical self-efficacy	Personal beliefs	Opening to new ideas and organizational change
		Professional ability	Criticizing and analyzing one’s own teaching practice
		Teaching level	The actual level of individual teaching practice
	Professional learning and active perception of reflection opportunities driven by personal goal orientation	Positive reflection	Motivated to participate in teaching reflection activities
		Professional study	Making continuous career advancement
		Development motivation	Willingness for self-improvement and development

## Data Availability

The data are not publicly available due to privacy restrictions.
